# Liberal Bequest

**Published:** 1856-05

**Authors:** 


*Liberal Bequest.*—The late Thomas Copeland, Esq., an eminent surgeon of London, who died in November last, has left the munificent sum of £5,000 to the Society for the Relief of Widows and Orphans of Medical Men. The personal property of the deceased was sworn under £180,000, the greater part of which was amassed by him in the practice of his profession.—*Boston Med. & Surg. Journal.*

